# Differentially hypomethylated cell-free DNA and coronary collateral circulation

**DOI:** 10.1186/s13148-022-01349-w

**Published:** 2022-11-01

**Authors:** Jongseong Ahn, Sunghoon Heo, Soo-jin Ahn, Duhee Bang, Sang-Hak Lee

**Affiliations:** 1 Department of Chemistry, Yonsei University, 50, Yonsei-ro, Seodaemun-gu, Seoul, 03722 Korea; 2 Integrative Research Center for Cerebrovascular and Cardiovascular Diseases, Yonsei University College of Medicine, 50-1, Yonsei-ro, Seodaemun-gu, Seoul, Korea; 3 Division of Cardiology, Severance Hospital, Yonsei University College of Medicine, 50-1, Yonsei-ro, Seodaemun-gu, Seoul, 03722 Korea; 4 Pohang University of Science and Technology (POSTECH), Pohang, Korea; 5 IMBdx, Seoul, Korea

**Keywords:** DNA methylation, Coronary artery disease, Cell-free DNA, Differentially methylated regions, Random forest, Biomarkers

## Abstract

**Background:**

The factors affecting cardioprotective collateral circulation are still incompletely understood. Recently, characteristics, such as CpG methylation of cell-free DNA (cfDNA), have been reported as markers with clinical utility. The aim of this study was to evaluate whether cfDNA methylation patterns are associated with the grade of coronary collateral circulation (CCC).

**Result:**

In this case–control study, clinical and angiographic data were obtained from 143 patients (mean age, 58 years, male 71%) with chronic total coronary occlusion. Enzymatic methyl-sequencing (EM-seq) libraries were prepared using the cfDNA extracted from the plasma. Data were processed to obtain the average methylation fraction (AMF) tables of genomic regions from which blacklisted regions were removed. Unsupervised analysis of the obtained AMF values showed that some of the changes in methylation were due to CCC. Through random forest preparation process, 256 differentially methylated region (DMR) candidates showing strong association with CCC were selected. A random forest classifier was then constructed, and the area under the curve of the receiver operating characteristic curve indicated an appropriate predictive function for CCC. Finally, 20 DMRs were identified to have significantly different AMF values between the good and poor CCC groups. Particularly, the good CCC group exhibited hypomethylated DMRs. Pathway analysis revealed five pathways, including TGF-beta signaling, to be associated with good CCC.

**Conclusion:**

These data have demonstrated that differential hypomethylation was identified in dozens of cfDNA regions in patients with good CCC. Our results support the clinical utility of noninvasively obtained epigenetic signatures for predicting collateral circulation in patients with vascular diseases.

## Background

The development and presence of coronary collateral circulation (CCC) has great clinical importance in patients with ischemic heart disease. Good CCC can reduce adverse cardiovascular events and infarct size when coronary arteries are occluded [1]. The involvement of growth factors, cytokines, and shear stress in collateral circulation development has been previously reported [1, 2]. However, the factors and predictors associated with collateral circulation are incompletely understood, with limited evidence on epigenetic impact. Human DNA methylation refers to the methylation of the C5 position of cytosine in CpG dinucleotides [3]. DNA methylation plays an important role in regulating transcription, embryonic development, genomic imprinting and stability, and chromatin structure. Thus, human diseases are often accompanied by changes in methylation patterns [4, 5]. Although the associations of DNA methylation with angiogenesis and vascular growth have been analyzed in mice [6, 7], related human studies have not been conducted. Cell-free DNA (cfDNA) refers to the circulating DNA released into the plasma through various mechanisms, including cell death [8]. The methylation patterns of organ-related cfDNA were recently detected in patients with sepsis and cancer [8]. Thus, researchers aim to aid diagnosis of disease like cancers based on the characteristics of noninvasively collected human cfDNA [9]. However, the clinical application of cfDNA methylation patterns remains limited as it is difficult to interpret due to its complex composition [8]. The amount of cfDNA is generally insufficient to maintain bisulfite conversion quality [9, 10], which is a gold standard in analysis. Fortunately, recent studies using enzymatic methyl-sequencing (EM-seq) presented promising results with limited cfDNA, using an enzymatic approach instead of harsh bisulfite conversion [11, 12]. Another study simplified the complex methylation patterns by introducing values such as the average methylation fraction (AMF) [9]. CpG methylation varies regionally according to the presence of adjacent CpG methylation and CpG density, enabling the implementation of these methods [13, 14]. These reports suggested using noninvasively obtained cfDNA to assess methylation characteristics, and its clinical use was also recently investigated [15, 16]. The aim of this study was to evaluate whether cfDNA methylation patterns are associated with the CCC status in patients with chronic total coronary occlusion [17]. In addition, the biological pathways associated with characteristic cfDNA methylation were analyzed. For the study, EM-seq and a series of data processing methods, including machine learning [18], were used.

## Result

### Unsupervised analyses reveal the methylation characteristics of cfDNA associated with CCC

**Fig. 1:**
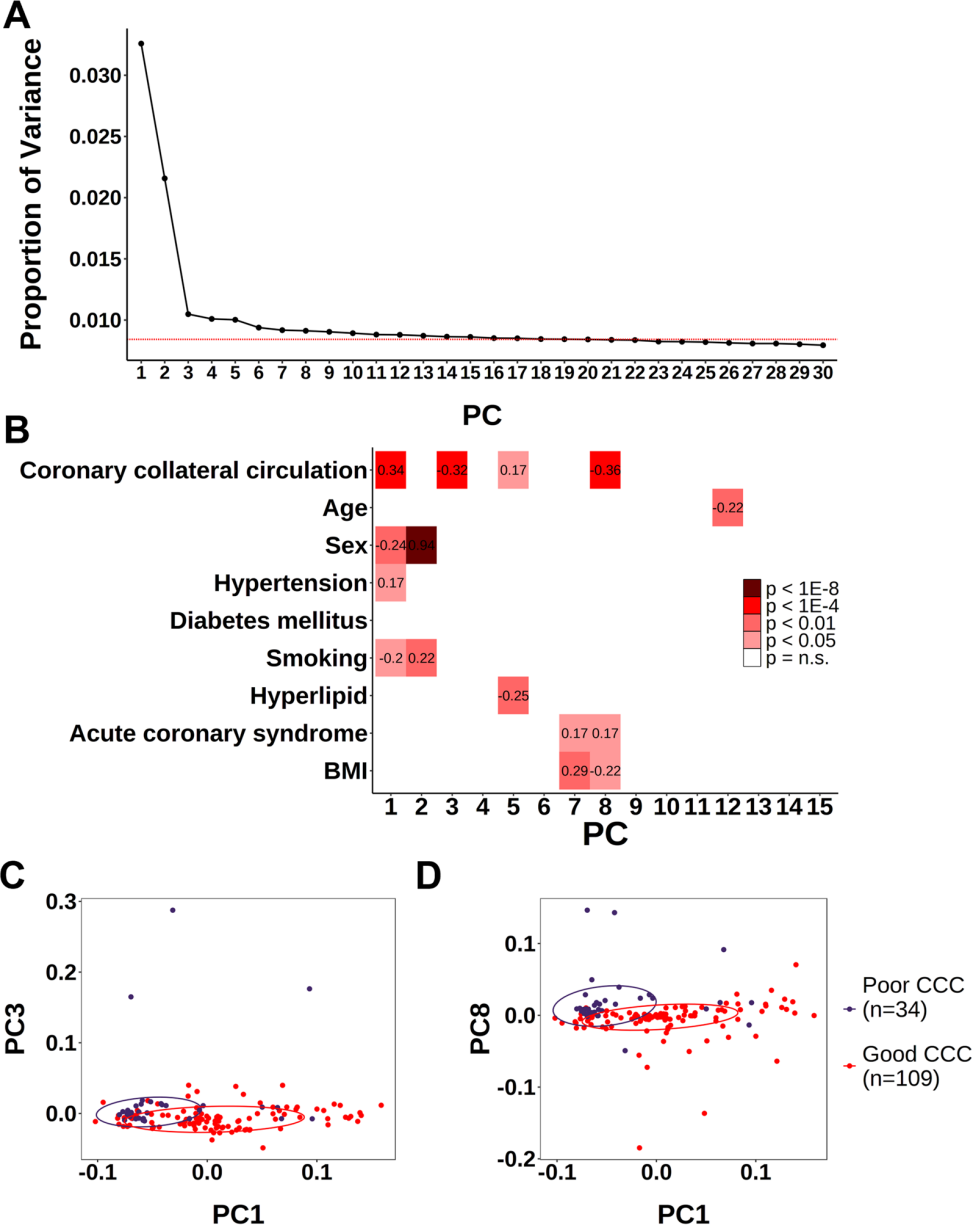
Correlation between DNA methylation and CCC. A PCA was conducted on data from all participants with good ($n=$ 109) and poor CCC ($n=$ 34). **A** Scree plot of the proportion of variance (y-axis) explained using the 30 PCs from a PCA on the AMF data table (black line). To determine effective PC values, the maximum value among the proportions of variance calculated from the random permutation AMF table is indicated using the red line; 15 PCs exist with observed variance higher than expected by the background. **B** Heatmap of the *p* values of associations between the 15 significant PCs and clinical variables. All *p* values were estimated with the Pearson’s correlation coefficient analysis. The numbers in each block represent the Pearson’s correlation coefficient. **C** PCA plots for PC1 and PC3 estimated to correlate with CCC. **D** PCA plots for PC1 and PC8 estimated to correlate with CCC

DNA methylation data were obtained in 143 patients (109 in the good CCC group and 34 in the poor CCC group) using EM-seq (Table 1). All samples passed the quality control process. No significant correlations were identified between CCC and clinical variables. As DNA methylation can be affected by various factors, we used PCA to identify the methylation characteristics. Rather than using the traditional subjective PC selection through the ‘elbow’ observation of the scree plot [19], we selected 15 PCs that exceeded the cutoff by estimating the maximum noise level [20, 21] (Fig. 1A). PC1 (Pearson’s correlation coefficient [PCC], 0.34), PC3 (PCC, $-0.32$), and PC8 (PCC, $-0.36$) showed significant (p < 1E-4) correlations with CCC (Fig. 1B). The correlations between PC and CCC were reproduced using the nonparametric method (Additional file 1: Fig. S1A). Based on these results, we confirmed that the distributions of PC1, PC3, and PC8 were associated with CCC. The PC1 samples of the good CCC group showed a wide distribution, whereas those of the poor CCC group showed a relatively narrow distribution (Fig. 1C). A few PC3 samples from the poor CCC group were outliers, with no differences in the overall distribution between the two groups (Fig. 1C). The overall distribution of the PC8 samples differed between the good and poor CCC groups, although with a much smaller distribution than that of the major component, PC1 (Fig. 1D). Differential component clustering was observed with the PCA of each group, despite the absence of pre-assigned labels. This was repeated in t-SNE using the same input values as the PCA (Additional file 1: Fig. S1B). The unsupervised analyses showed that methylation characteristics were associated with CCC. Furthermore, differential methylation was observed in cfDNA.

**Table 1:** Characteristics of the study participants

	Total	Good CCC	Poor CCC	
	(*n* = 143)	(*n* = 109)	(*n* = 34)	*p*
Age, years	57.8 ± 10.6	57.0 ± 11.2	59.7 ± 9.9	0.25
Male	102 (71.3)	78 (71.6)	24 (70.6)	>0.99
Risk factors				
Hypertension	68 (47.6)	54 (49.5)	14 (41.2)	0.44
Diabetes mellitus	34 (23.8)	26 (23.9)	8 (23.5)	>0.99
Smoking	19 (13.3)	13 (11.9)	6 (17.6)	0.39
Hypercholesterolemia	11 (7.7)	8 (7.3)	3 (8.8)	0.72
Acute coronary syndrome	89 (62.2)	65 (59.6)	24 (70.6)	0.31
Body mass index, kg/$m^2$	25.2 ± 3.0	25.3 ± 3.1	24.6 ± 3.0	0.31
*Number of diseased vessels*				
1	49 (34.3)	38 (34.9)	11 (32.4)	
2	30 (21.0)	24 (22.0)	6 (17.6)	0.82
3	64 (44.8)	46 (42.2)	17 (50.0)	

Data are presented as the mean ± standard deviation or number (%)

### DMR search identifies predominant hypomethylation, while the filtered DMRs show reproducibility of grouping in CCC

**Fig. 2:**
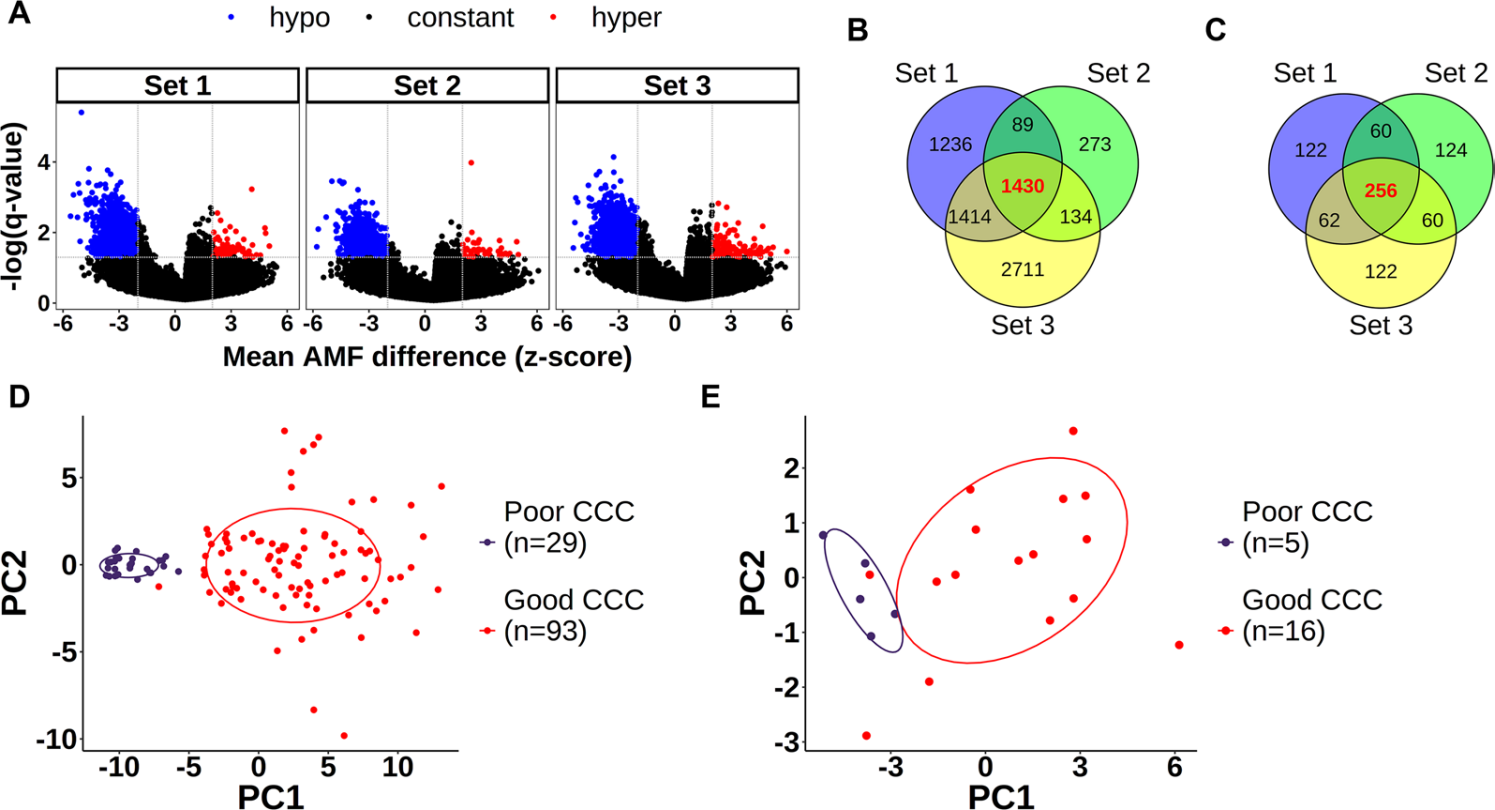
Screening process for DMRs potentially associated with CCC. Selection of DMR candidates in each of the three subsets sampled by replacement from the training sets. **A** Volcano plots examining associations between mean differences in AMF and *q* values. *p* values are calculated using Welch’s t-test on 600,000 bins for which >90% AMF values could be calculated in each subset. Negative log-transformed *q* values generated using FDR correction of *p* values plotted for the differences in AMF between good and poor CCC groups (converted to z-scores via standardization). The area above the dashed horizontal line indicates *q* values <0.05. The dashed vertical line indicates the absolute z-score value |*z*| of 2. When the mean AMF value is significantly lower in the good CCC group than in the poor CCC group, it is indicated as hypomethylation (blue). Conversely, a high mean AMF value is indicated as hypermethylation (red). **B** DMRs selected from each of the three subsets. A total of 1430 DMRs commonly included in the three subsets were used in additional filtering processes. **C** Top 500 DMRs in each subset selected from 1430 DMRs based on the significance of the *q*-value. A total of 256 DMRs included in top 500 of all subsets were selected as input values for the additional random forest classifier step. **D** PCA results of the 256 DMRs using the AMF values of all training set samples (poor CCC: $n=$ 29; good CCC: $n=$ 93). **E** PCA results of the 256 DMRs using the AMF values of the test set samples (poor CCC: $n=$ 5; good CCC: $n=$ 16). The PCA results of the test set were predicted using the learning test results based on the training set

We selected methylation marker regions that could be used to predict good CCC. We first reduced the number of variables through pre-screening prior to marker screening using machine learning. This was based on a previous observation that an increase in unnecessary variables lowers prediction accuracy [22]. The pre-screening process consisted of CCC-associated DMR screening and the screening of candidate markers among the selected DMRs. The training and test sets were divided for verification, and screening was only performed in the training set. DMR searches were conducted for each of the three resampled subsets in the CCC-related DMR detection training set to prevent overfitting. The difference in means between the good and poor CCC groups in the same bin appeared to be a mixture of two aspects (Additional file 1: Fig. S2). Methylation differences were observed in some bins, and hypomethylation tended to be more common. Bins with significantly differing AMF distributions between the good and poor CCC groups were selected using Welch’s t-test (Additional file 1: Fig. S3). As significant methylation differences between the two groups were observed in the unsupervised analysis, we assumed that the Welch’s t-test result was significant and conducted the subsequent analysis. DMRs were selected based on the differences in means and distributions (Fig. 2A). Hypomethylation (z-score $< -$2) was more common and more variable than hypermethylation (z-score > 2) in the selected DMRs. Although the number of hypomethylated and hypermethylated DMRs was different in each set, the predominance of hypomethylation and low CpG density was consistent (Additional file 1: Fig. S4). Thereafter, we selected the most reproducible DMRs from those identified in each subset. Only DMRs observed in all three subsets were selected in the screening process for reproducible DMRs that were not sample-specific, and 1430 DMRs met this criterion (Fig. 2B). The 1430 intersection DMRs were ordered according to the *q* values of each subset. Pathway analysis of the 1430 DMRs did not (1) reveal a clear association with previously known CCC-related pathways, or (2) match the predicted results based on other databases (Additional file 1: Fig. S5). Only 256 in the top 500 DMRs in all subsets were selected as candidate marker DMRs strongly associated with CCC (Fig. 2C). Pathway analysis of the 256 DMRs identified factors reported to be related to CCC, including TGF-beta, G-protein, and eosinophils (Additional file 1: Fig. S6) [23–25]. The PCA of the entire training set using 256 selected DMRs identified separation that depended on the CCC group (Fig. 2D). This confirmed the potential of the selected 256 DMRs to predict good CCC. PCA prediction was then performed by replacing the input data with a test set not used for DMR screening. Group clustering was observed, although with some overlap (Fig. 2E). Taken together, these 256 candidate DMRs demonstrated the potential to be used as universal CCC markers rather than overfitting the training set.

### Random forest classifier selects the marker DMRs of CCC

**Fig. 3:**
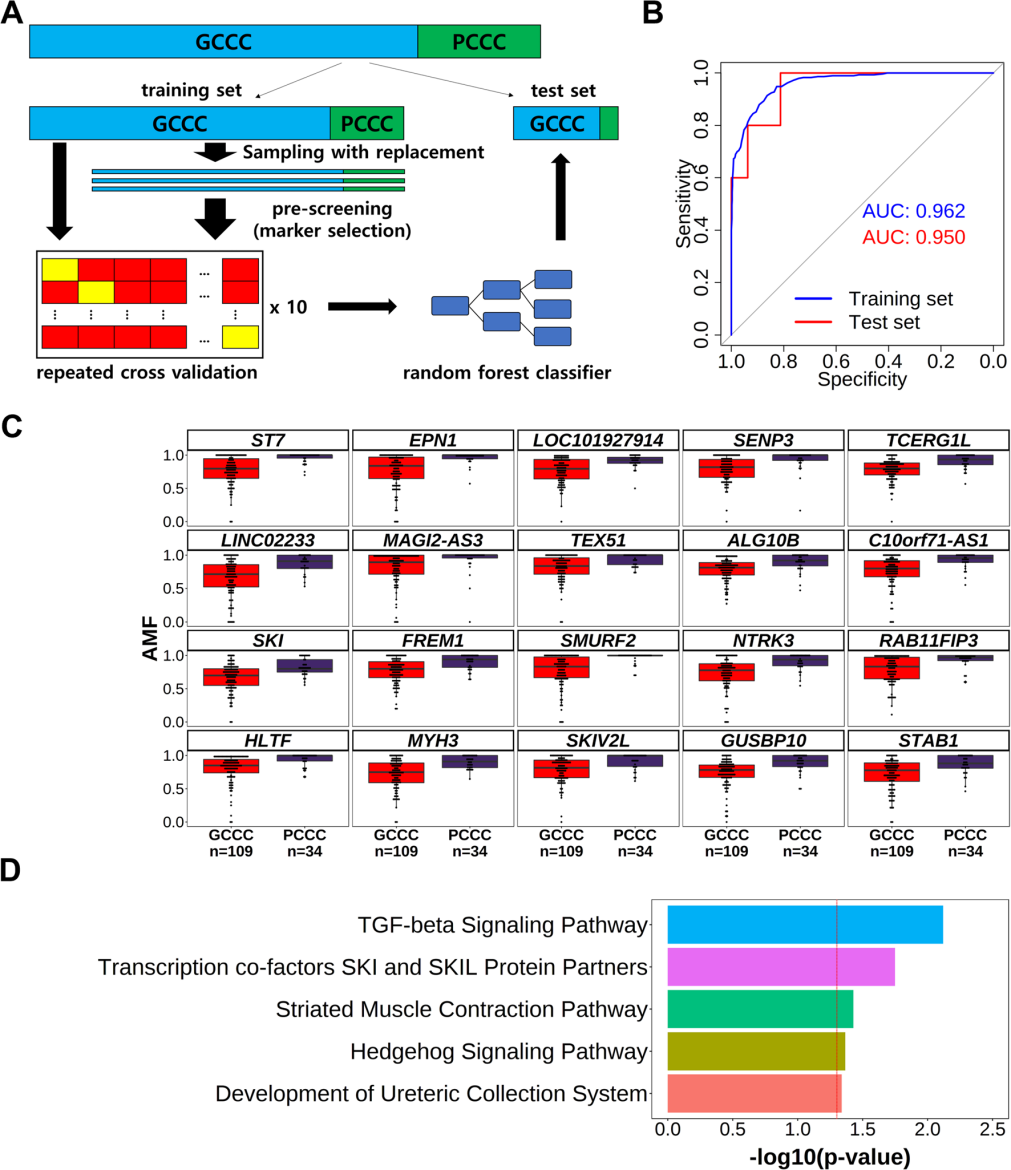
Selection of major DMRs related to good CCC using a random forest classifier. **A** Flowchart of random forest classifier training and validation through repeated cross-validation using good CCC-related DMR candidates. For the entire training set (poor CCC: $n=$ 29; good CCC: $n=$ 93), AMF values of 256 pre-screened CCC-related DMR candidates were used for training. To validate the training results, predictions were made on pre-separated test set samples (poor CCC: $n=$ 5; good CCC: $n=$ 16). **B** ROC curves for the learning test results of the training set (blue) and the prediction results of the test set (red). **C** A box plot of the AMF distribution and annotations of good (red: $n=$ 109) and poor CCC (violet: $n=$ 34) groups. These correspond to the top 20 DMRs determined from the random forest classifier training results. **D** Pathway analysis results of the 20 DMRs associated with CCC

Finally, we performed marker selection using machine learning. We trained a classifier using the random forest method of learning algorithms and used the 256 selected DMRs as input. Repeated cross-validation was performed using the training set (Fig. 3A). The prediction performance of the classifier was measured using the AUC of the ROC curve for the test set (Fig. 3B). The AUC values of the test set resembled those of the training set, and we assumed that the measured importance was valid. Based on the importance given in the final model, we selected the top 20 DMRs as markers and evaluated the AMF distribution of each DMR (Fig. 3C). Among these 20 DMRs, five were located in the exon region, eight in the intron region, and seven in the intergenic region. The poor CCC group generally showed a narrow distribution of AMF values close to 1, whereas the good CCC group presented a wide AMF distribution. This AMF pattern suggested that the selected DMRs showed differences in methylation and were suitable as markers for good CCC. We further performed a pathway analysis to investigate the biological relevance of the 20 selected marker DMRs (Fig. 3D, Additional file 1: Fig. S7). In all databases, the TGF-beta-associated pathway was repeatedly observed to be associated with selected DMRs. These results prove that the association observed between the selected DMRs and the TGF-beta pathway was not biased by the database, a well-known problem [26]. Finally, we validated our selected markers using data from a public dataset. We have obtained CpG methylation data from previously published cfDNA from healthy subjects [27]. We evaluated the AMF distribution at the remaining 18 DMRs, with the exception of two DMRs not included in the publication data (Additional file 1: Fig. S8). In all 18 DMRs, the AMF distribution in the healthy group was similar to or more stringent than that of the poor CCC group. These observations support that hypomethylation of our selected markers is CCC-specific.

## Discussion

The major findings of the present study include the following: (1) EM-se*q*-based methylation profiling produced good quality data, even with limited human cfDNA quantities; (2) samples from patients with good CCC exhibited a wide distribution of AMF in selected DMRs, whereas those from patients with poor CCC presented a narrow distribution; (3) distinct CpG methylation of human DNA associated with good or poor CCC was identified and validated using this noninvasive cfDNA analysis method, where patients with good CCC presented predominantly hypomethylated cfDNA; and (4) the identification of pathways, such as TGF-beta signaling, that were associated with selected DMRs indicated the biological relevance of the marker DMRs. Taken together, these results suggest the utility of cfDNA methylation as a predictive tool for cardiovascular conditions such as collateral circulation.

Until recently, similar bisulfite conversion studies required relatively large amounts of DNA due to DNA degradation. Of note, this study was performed using limited cfDNA quantities obtained using a noninvasive approach. Maintaining data quality with the recently introduced EM-seq method [12] contributed to our results. We performed unsupervised and supervised analyses separately to avoid confirmation bias. In both assays, differences in DNA methylation were clearly observed between the two CCC groups. Comparison with published cfDNA data from healthy subjects also supports that our selected markers are specific to good CCC. The broad AMF distribution of the good CCC group and the narrow AMF distribution of the healthy group are interesting observations, considering that the cfDNA data were obtained from a combination of multiple sources. A recent murine study revealed that DNA methyltransferase 1-dependent endothelial DNA methylation constrains arteriogenic capacity [27]. Thus, DNA methylation could impact collateral circulation that was verified in human samples for the first time in the current study. Another recent study analyzed DNA methylation in human carotid plaques, but could not replicate the differential methylation of shear stress-associated genes discovered in mouse models [28]. Therefore, our results on human samples provide rare evidence of distinct DNA methylation-associated vascular conditions.

Among the selected DMRs, SKI [29] and SMURF2 [30] are directly related to TGF-beta, and their hypomethylation may also be biologically relevant to CCC development. Various DMRs were located in the intron regions of several genes, including the two aforementioned genes. Differential methylation in introns can affect specific genes and change their expression [31]. Several other genes with DMRs identified in our analyses showed potential biological relevance in CCC. Epsin1, encoded by EPN1, is related to angiogenesis in cancer patients [32]. SENP3, which encodes a redox-sensitive enzyme, mediates vascular remodeling [33].

The 20 marker DMRs identified in the present study showed common associations with the TGF-beta pathway in multiple databases. TGF-beta expression is induced under hypoxic conditions and it mediates angiogenesis in infarcted hearts [34]. This signaling pathway increases the expression of an endothelial receptor and contributes to vascular structural change [35]. Thus, the association of TGF-beta signaling in our analyses may indicate a plausible CCC-promoting mechanism. In addition, G-protein [25] and eosinophil [24] pathways found in DMR screening (Additional file 1: Fig. S5) are associated with arteriogenesis or collateral circulation. Abundance of a G-protein signaling-related protein was previously found to be increased in the vascular smooth muscle cells of collateral arterioles [25]. Conversely, the eosinophil count independently predicted high-grade CCC in individuals with unstable angina [24]. Thus, the DMRs identified in the present study could be linked to biological pathways related to CCC. Further studies and verification of their biological relevance may strengthen the implications of our results.

Our study had several limitations. We used AMF values of bins with high CpG densities for reproducibility at low-sequencing depths. In addition, the limited number of patients with poor CCC might have biased the patient grouping used for machine learning. When the sample size is large enough, a simple comparison is often sufficient. However, in our case the sample size was not sufficient to correct for bias, leading to overfitting with a simple comparison (data not shown). Therefore, we tried to discover valid markers by verifying reproducibility in a separate test set after training and validation. A larger sample size might have helped enhance the value of the validation results; therefore, cross-validation with external study populations or other experimental methods should be used in future studies to increase reliability. Importantly, despite these limitations, our study is significant due to the novelty of being the first to identify distinct methylation of cfDNA predicting good CCC using human samples obtained with noninvasive methods.

## Conclusion

In conclusion, distinct CpG methylation dependent on the CCC grade was identified in human cfDNA. The association between biological pathways including TGF-beta signaling and selected DMRs, indicated the biological relevance of these methylated regions. These results suggest the utility of differential cfDNA methylation as a predictor of cardiovascular conditions, such as collateral circulation.

## Methods

### Study population and clinical data collection

All patients included in this study visited the Severance Hospital from January 2001 to August 2009 and received coronary angiography for chest discomfort or pain [36]. We used the patient data deposited in the Cardiovascular Genome Center database of the Yonsei University College of Medicine, Korea. Patients who presented with chronic total occlusion of at least one epicardial coronary artery were selected for this study. This study conformed to the Declaration of Helsinki and obtained approval from the Institutional Review Board of Severance Hospital, Seoul, Korea (4-2019-0880). Trained nurses collected clinical data, including demographic variables and risk factors. Blood samples were obtained from all study subjects immediately before or within 24 h post-angiography and stored at -80$^{\circ }$C. The majority of patients had blood drawn before angiography, and only a few patients had blood collected afterward. Patients were given oral aspirin and 5,000 U of intravenous heparin, followed by angiography. Coronary artery disease and CCC were confirmed by two interventional cardiologists, who were blinded to other patient data. CCC was assessed according to the Rentrop classification: grade 0, no filling; grade 1, filling of the side branches via the collateral channels without epicardial filling; grade 2, partial filling of the epicardial coronary artery via the collateral channels; and grade 3, complete filling of the epicardial coronary artery [37]. Patients were classified based on the collateral grades as having poor (grade 0 or 1) or good (grade 2 or 3) CCC.

### Cell-free DNA preparation and EM-seq library production

cfDNA was extracted from plasma using the QIAamp MinElute ccfDNA Kit (Qiagen, Hilden, Germany) and stored at $-20$ $^{\circ }$C. The cfDNA concentrations and size distributions were assessed using TapeStation (Agilent, Santa Clara, CA, USA) before library preparation. EM-seq libraries were prepared using 1-100 ng of cfDNA and an EM-seq kit (New England Biolabs, Ipswich, MA, USA) without fragmentation. Library concentrations and distributions were also determined using TapeStation. Paired-end 150-bp sequencing was performed using the NovaSeq 6000 S4 platform (Illumina).

### Data preprocessing and average methylation fraction (AMF) table creation

All sequencing data were trimmed using fastp (version 0.20.1) [38]. Adapter-trimmed reads were aligned onto the hg19 reference genome using bitmapperBS (version 1.0.2.3) [39]. The output bam file was sorted using Samtools (version 1.11) [40]. PCR and optical duplicates were removed using the GATK (version 4.1.9.0) MarkDuplicates module [41] (Additional file 2: Table S1). The blacklisted genomic regions in ENCODE [42] and the repeat element regions screened using RepeatMasker (http://www.repeatmasker.org) were obtained to remove alignment artifacts. Reads overlapping these regions were filtered out prior to the analysis. A final, filtered BAM file was used to calculate the methylation levels at each cytosine locus using MethylDackel (https://github.com/dpryan79/MethylDackel). The conversion rate was calculated with an in-house Python program (version 2.7.17) using the MethylDackel CHH output as the input. Samples were excluded if the conversion rate did not exceed 99% or if the median of average depth was < 3. The hg19 reference genome was partitioned into 100-bp bins for all regions. AMF values were obtained for the filtered BAM file for each sample in  1.2 million bins with high CpG density containing five or more CpGs to increase reliability at a low-read depth. AMF was defined based on a previous report [9] as follows: AMF is the ratio of the number of methylated CpG among all the aligned bin reads at known CpG positions in the reference genome. AMF values of each bin were obtained, and the samples were divided into good and poor CCC groups. Only bins with null values of < 10% were selected. A total of 606,483 bins fit the criteria and were used for subsequent analyses. The table is publicly accessible at https://osf.io/fw2zq. The above process was performed using R (version 4.0.3).

### Unsupervised analysis

The standard deviation (SD) of each bin was calculated using the AMF table. The SD was standardized and bins with a z-score of > 2 were selected, excluding background fluctuations. A total of 42,092 bin positions were selected. The missing values were replaced with the means of the good and poor CCC groups in which they were categorized. Principal component analysis (PCA) was also performed; PCA of large-sized tables was performed using R package flashpcaR (version 2.1) [43]. The PCA values of the top 30 components were calculated by setting *k* = 30. To obtain effective principal components (PCs), samples were randomly shuffled 1000 times. The maximum total variance obtained through the PCA of 1000 shuffled tables was considered the effective PC cutoff. The selected 15 PCs were analyzed for correlations with clinical variables. The Rtsne package (https://github.com/jkrijthe/Rtsne) was used for t-SNE analysis. The Pearson and Spearman correlation coefficients and *p* values between the PC values and clinical variables were calculated. Categorical variables were converted to 0 and 1 and then computed as point-biserial correlation coefficients. The cor.test function in R was used for all calculations.

### Differentially methylated region (DMR) selection

For the previously constructed AMF table with bins containing < 10% missing values, samples were partitioned into training and test sets at a ratio of 85:15. Set separation was performed using the createDataPartition function included in the caret package (version 6.0.86) [44] in R. The Welch’s unequal variances t-test [45] was applied to retrieve CCC-associated DMRs because of the heteroscedasticity of DNA methylation variance according to the genomic position [3]. Welch’s t-test was performed on each of the three sampled subsets sampled with replacement from training set to select bins with AMF differences between good and poor CCC groups. The actual process was performed using the row_t_welch function in the matrixTests package (https://github.com/karoliskoncevicius/,version 0.1.9) in R. The results included the mean differences and *p* values between the two groups for each bin. The *p* value was converted to a *q* value using the R package fdrtool (version 1.2.16) [46]. Bins that satisfied the absolute value of the mean difference |*z*| > 2 and *q* value < 0.05 were selected as DMRs. The intersection of the DMRs in each of three subsets was found, and 1430 shared DMRs were confirmed. The rankings of the *q* values in each subset were then considered. The 1430 DMRs were sorted based on the *q* values calculated in each subset. Only the top 500 DMRs in all subsets were selected, with a final selection of 256 DMRs. A PCA of the training and test sets in the 256 DMRs was performed using the prcomp function in R with a 70% confidence interval to draw the core region.

### Random forest process

The full training set of 256 DMRs was used as input for the random forest analysis. Cross-validation of the training and validation sets was performed using the ‘repeatedcv’ option of the trainingControl function in the caret package. A tenfold cross-validation was repeated 10 times. Random forest classifier construction was performed using the caret train function by selecting the ‘rf’ option. The prediction effect of the model on the training and test sets was evaluated using the area under the curve (AUC) of the receiver operating characteristic (ROC) curve. The optimal ROC curve was selected using the pROC (version 1.17.0.1) package [47] in R to calculate the corresponding specificity and sensitivity. The importance of individual variables was evaluated based on the ‘MeanDecreaseGini’ value in the importance of the final constructed random forest model.

### Annotation and pathway analysis

A list of DMRs for pathway analysis was created in BED format using R. The DMR-related gene list was created using HOMER (version 4.11) genomic annotation [48]. Deduplication was performed, and a list of related genes was inputted into Enrichr (https://maayanlab.cloud/Enrichr/) [49] for pathway analysis. Primary results based on the WikiPathways 2021 [50] and the Elsevier pathway and Panther 2016 [51] databases were further considered.

### Comparison of AMF patterns with healthy human cfDNA in screening DMRs

Healthy human cfDNA data from previous published papers [52] were downloaded from the GEO database (GSE164600) in bed file format. The bed file lists the number of mappings and methylations at individual CpG locations. The AMF was obtained as previously described by filtering the CpG information overlapping with the previously selected DMR using the bedtools (version 2.29.2) intersect function. Of the total 12 healthy human cfDNA datasets, 11 patients were included, excluding one with very low coverage. Of the 20 DMRs, 2 DMRs were not covered, and the values in the remaining 18 DMRs were compared with the AMF distributions of the good CCC and poor CCC groups.

## Supplementary information

**Additional file 1**. Supplementary figures: figure S1-S8

**Additional file 2: Table S1**: Sheet 1—Summary of markduplicate results. Sheet 2—Summary of sample depth

## Data Availability

The AMF tables used directly in the analysis are publicly accessible at https://osf.io/fw2zq. The cfDNA methylation data from healthy individuals used for validation are available from GEO at http://www.ncbi.nlm.nih.gov/geo/ under Accession Number GSE164600 [52]. Raw whole-genome methylation sequencing datasets are provided upon reasonable request from the corresponding author (D.B.) upon review and approval by the institutional review board.
