# Supplementary material for: Differentially hypomethylated cell-free DNA and coronary collateral circulation
Source: Clin Epigenetics. 2022 Nov 1;14:140. doi: 10.1186/s13148-022-01349-w (PMC9628091; doi:10.1186/s13148-022-01349-w)
Supplement: Supplementary file 1 — Additional file 1. Supplementary figures: figure S1-S8 [file 13148_2022_1349_MOESM1_ESM.docx]

**Supplementary Figures**

PC: principal component

CCC: coronary collateral circulation

PCA: principal component analysis

AMF: average methylation fraction

FDR: false discovery rate

DMR: differentially methylated region

**
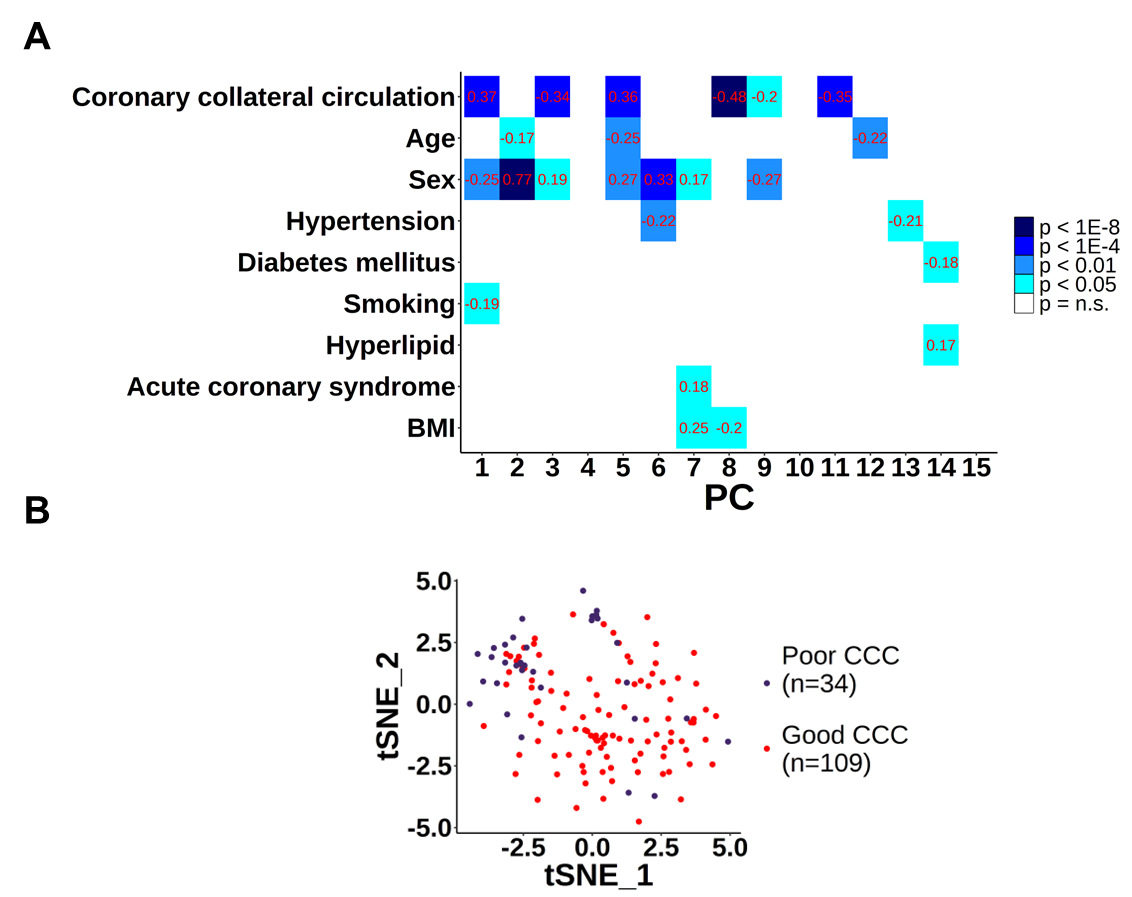
**

**Supplementary Figure S1. Confirmation of the unsupervised analytical results**. (A) Heatmap of the *p*-values of associations between the 15 significant PCs and clinical variables. All *p*-values were estimated using Spearman’s correlation coefficient analysis. The numbers in each block represent the Spearman’s correlation coefficient value. (B) Results of the t-SNE analysis using AMF values of bins with high variance as the input. The same PCA input values were used.

**
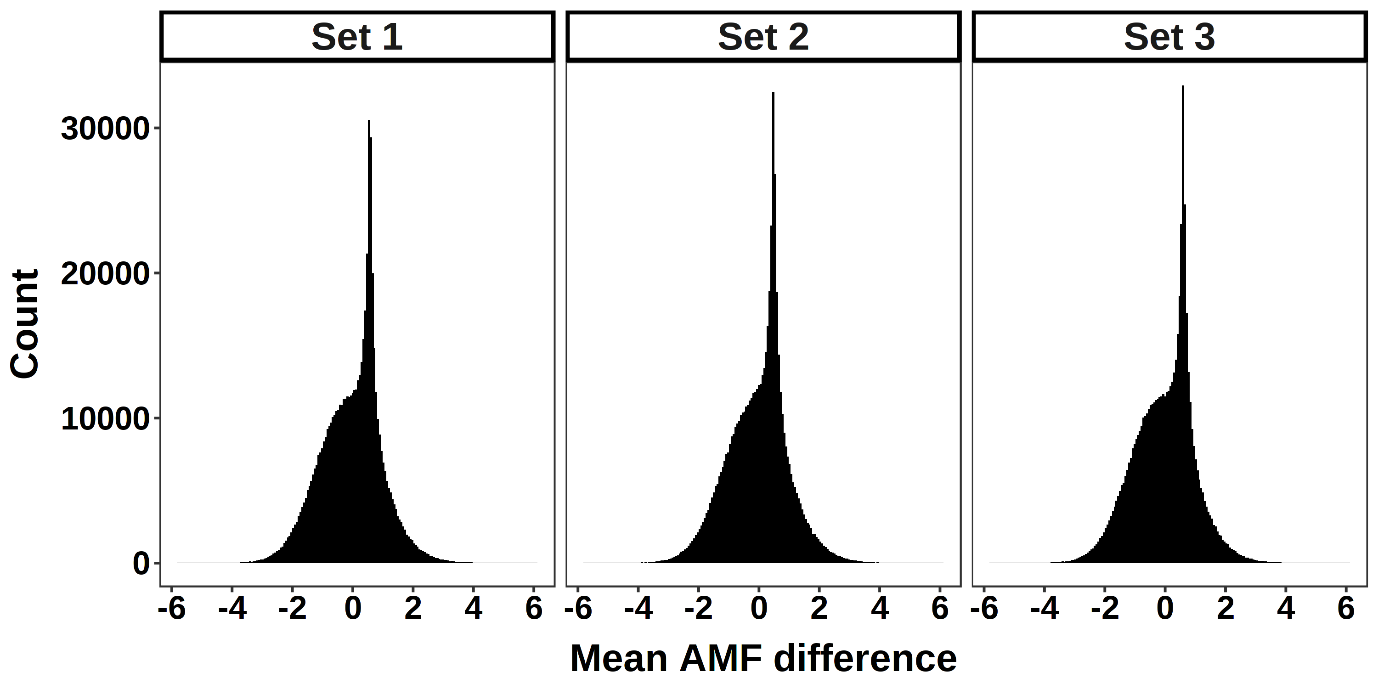
**

**Supplementary Figure S2. Distribution of mean AMF differences between the poor and good CCC groups.** x-axis: standard deviation of mean difference. y-axis: number of corresponding bins. A predominance of hypomethylation is observed when CCC occurs, so the peak indicating zero change in the mean is slightly biased toward hypermethylation.

**
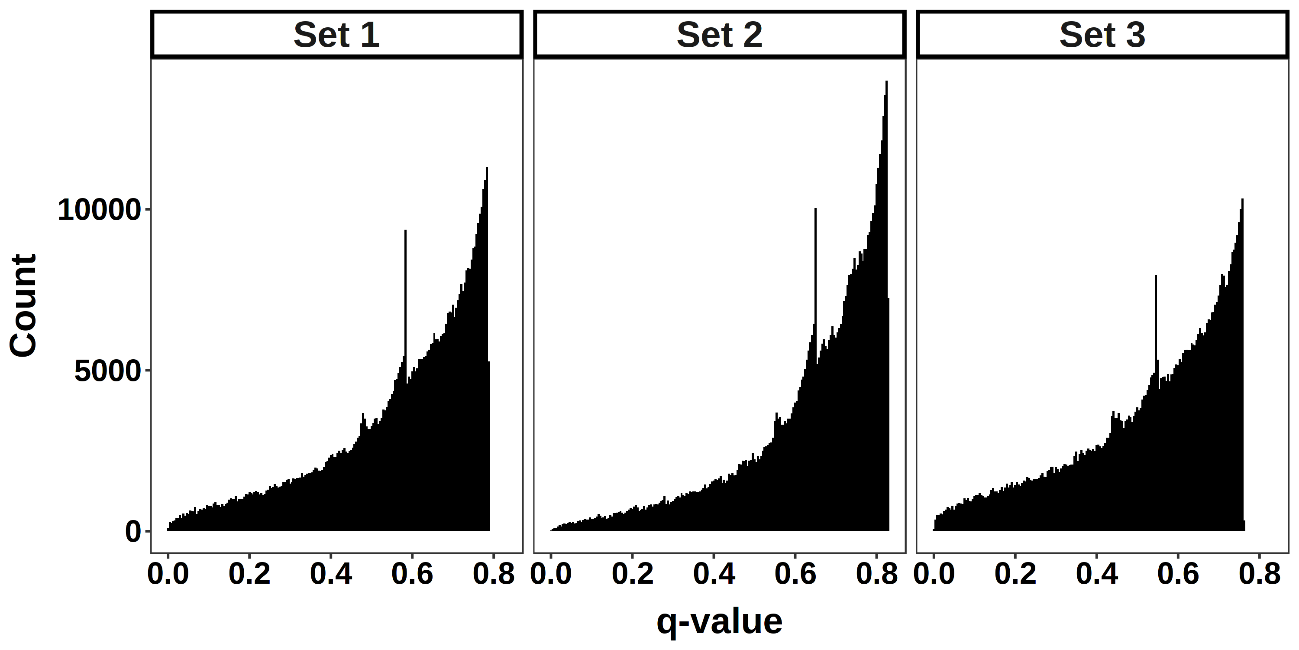
**

**Supplementary Figure S3. Distribution of q-values in each of the three resampled groups.** FDR correction applied to the *p*-value obtained from the Welch's t-test. The distribution of q-values in each of the three resampled groups is shown.


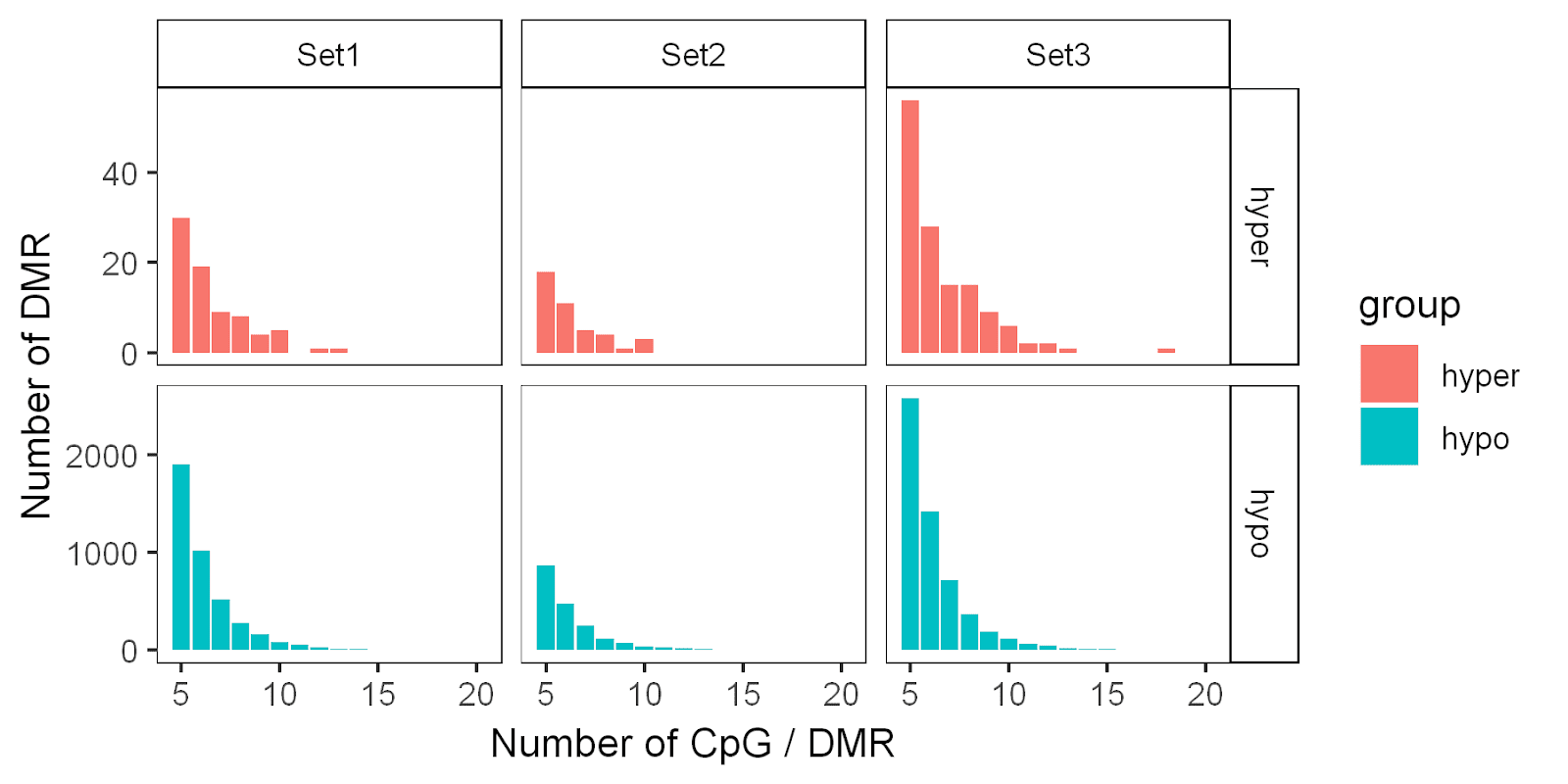


**Supplementary Figure S4. Distribution of CpG density in each of the three resampled groups.** The number of CpGs in an individual DMR is defined as the number of CpGs in the reference genome. As only regions containing 5 or more CpGs in individual bins were screened in our study, 5 CpG/DMR was the smallest bin included in the analysis.
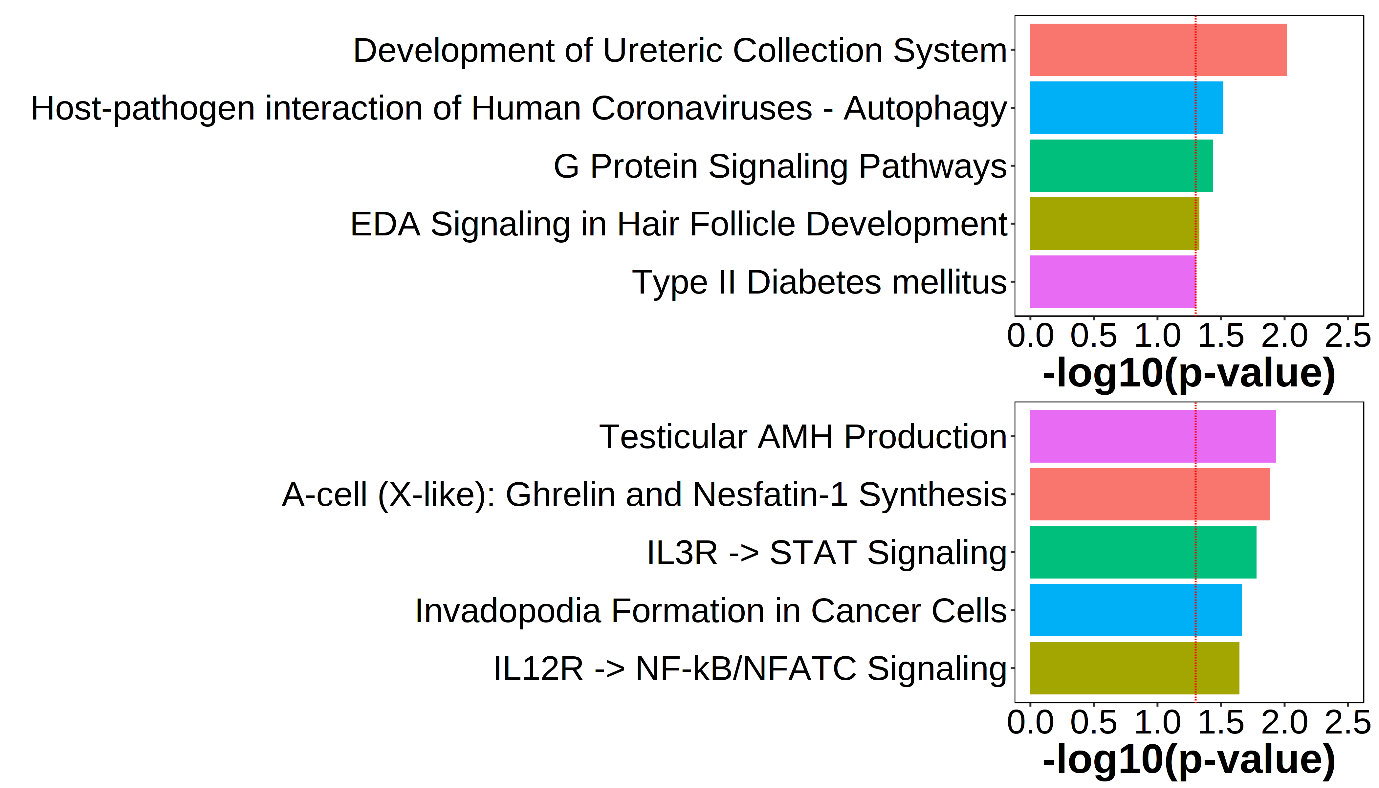


**Supplementary Figure S5. Pathway analysis results of the 1,430 DMRs, which are intersections of DMRs from the three subsets.** Upper panel: WikiPathway 2021; lower panel: Elsevier pathway. Red line: p = 0.05

**
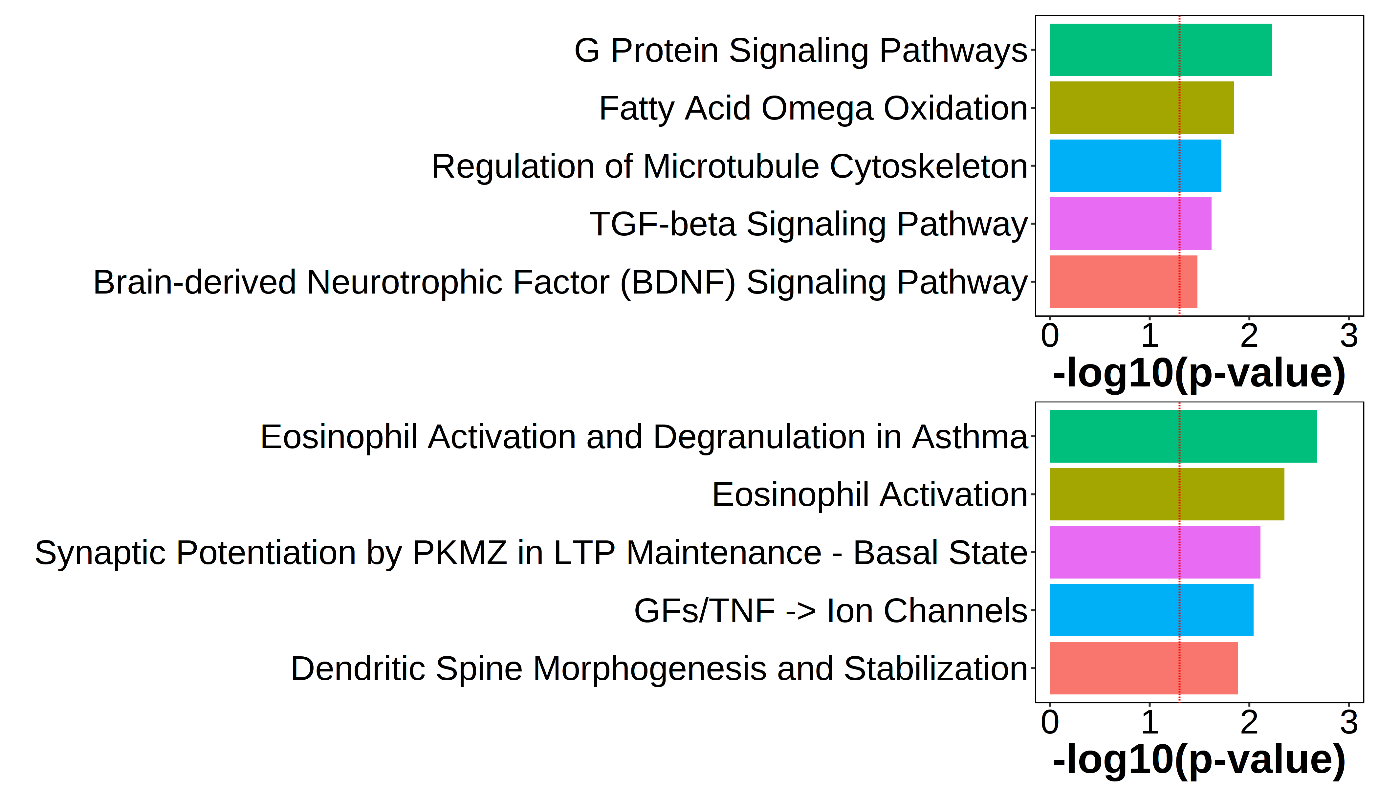
**

**Supplementary Figure S6. Pathway analysis results of the 256 DMRs selected from 1,430 intersection DMRs.** Upper panel: WikiPathway 2021; lower panel: Elsevier pathway. Red line: p = 0.05


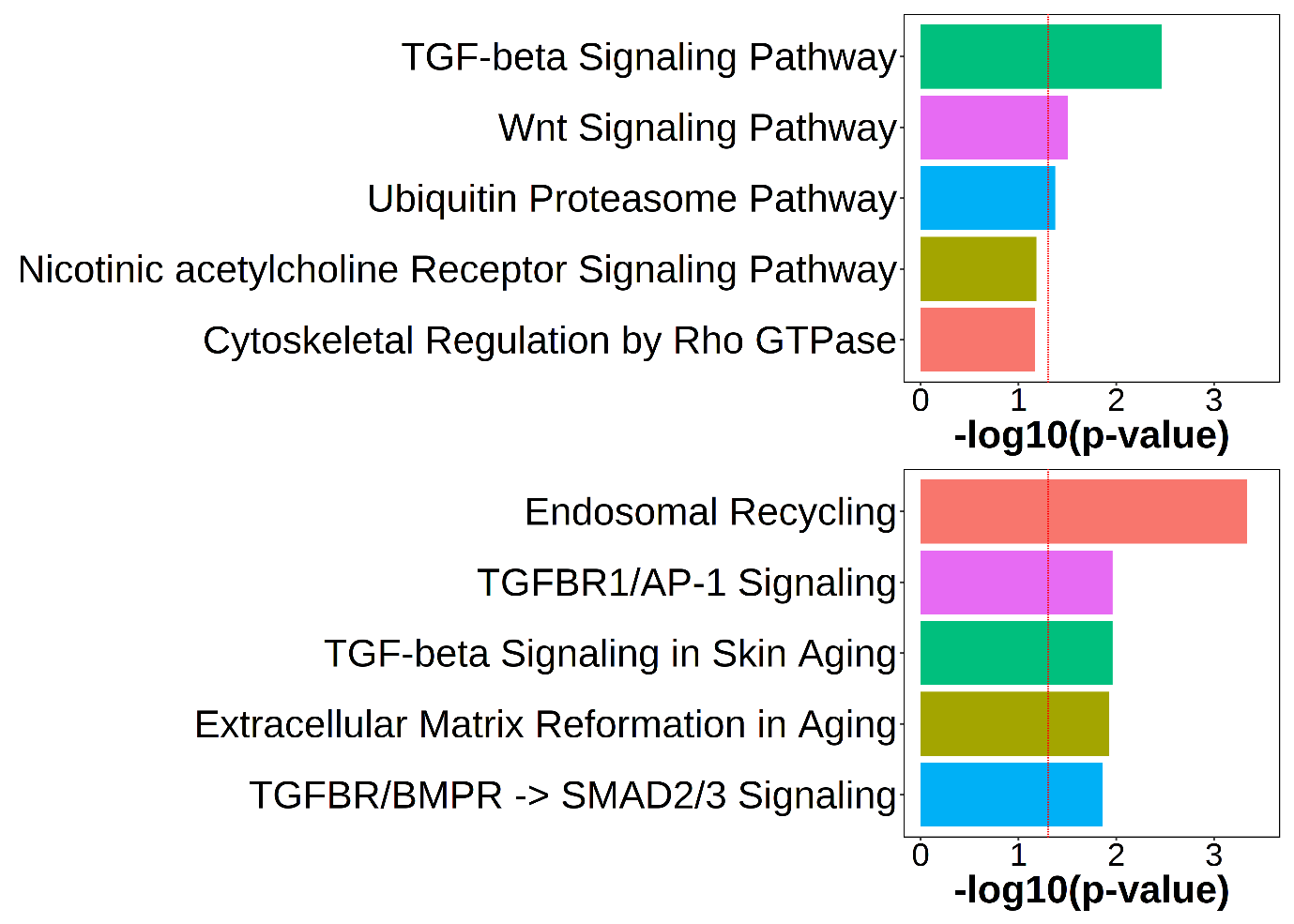


**Supplementary Figure S7. Pathway analysis results of the 20 important DMRs selected using a random forest classifier.** Upper panel: Panthers 2016; lower panel: Elsevier pathway. Red line: p = 0.05


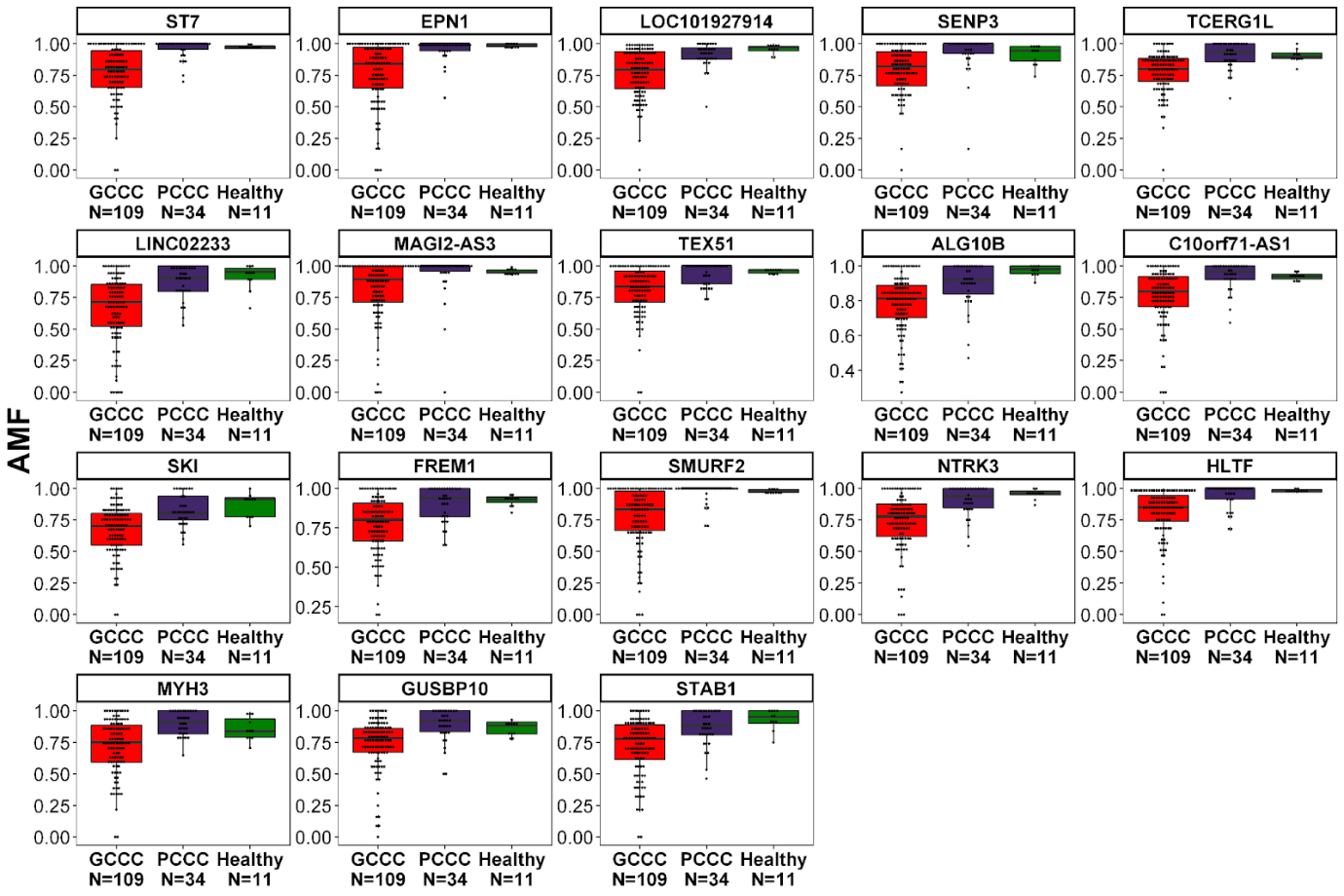


**Supplementary Figure S8. Comparison of AMF values between healthy and CCC groups in selected DMRs.** Among the selected 20 DMRs, the distribution of AMFs for each of the good CCC (red), poor CCC (violet), and healthy (green) groups in 18 DMRs mapped even in the healthy group was expressed as a boxplot.
